# Supplementary material for: Brief wide-field photostimuli evoke and modulate oscillatory reverberating activity in cortical networks
Source: Sci Rep. 2016 Apr 21;6:24701. doi: 10.1038/srep24701 (PMC4838830; doi:10.1038/srep24701)
Supplement: Supplementary Information [file srep24701-s1.pdf]

Brief wide-field photostimuli evoke and modulate oscillatory reverberating activity in cortical networks

Short title: Photo-activated network responses in vitro

Rocco Pulizzi[1], Gabriele Musumeci[1], Chris Van Den Haute[2-3], Sebastiaan Van De Vijver[1], Veerle Baekelandt[2], Michele Giugliano[1, 4-5]

[1] *Theoretical Neurobiology & Neuroengineering, University of Antwerp, Antwerp, Belgium;*
[2] *Laboratory of Neurobiology and Gene Therapy, Katholieke Universiteit Leuven, Leuven, Belgium;*
[3] *Leuven Viral Vector Core, Katholieke Universiteit Leuven, Leuven, Belgium*
[4] *Department of Computer Science, University of Sheffield, S1 4DP Sheffield, UK*
[5] *Laboratory of Neural Microcircuitry, Brain Mind Institute, EPFL, CH-1015 Lausanne, Switzerland*

Supplementary Information

- **Supplementary Figure S1**

- **Supplementary Figure S2**

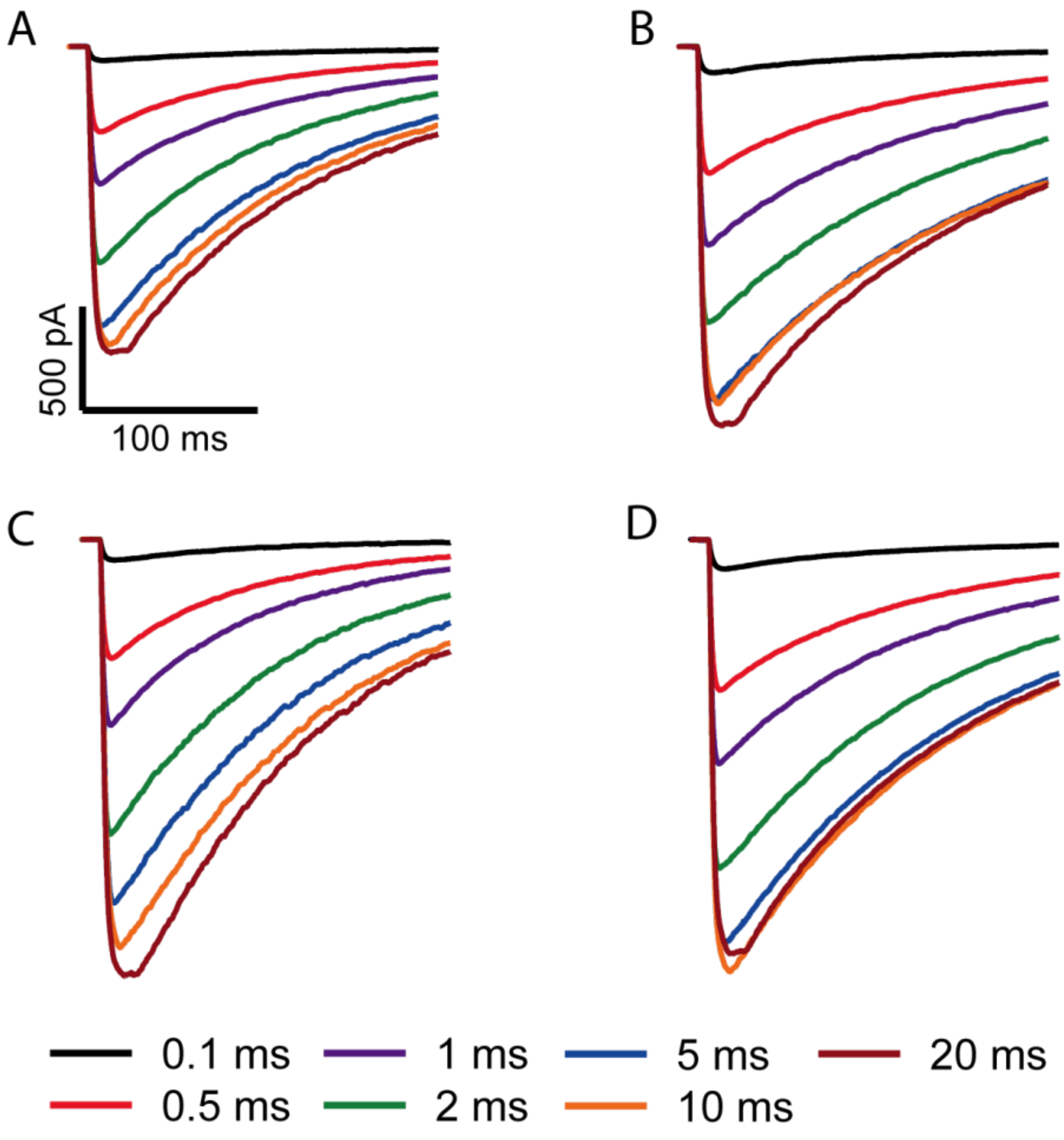

Figure S1. ChR2-LCTC current dynamics. Voltage-clamp experiments were performed under TTX in neurons expressing ChR2-LCTC, in order to explore the inward current dynamics. Varying the stimulus duration modulates the peak current amplitude in four representative neurons (**A-D**).

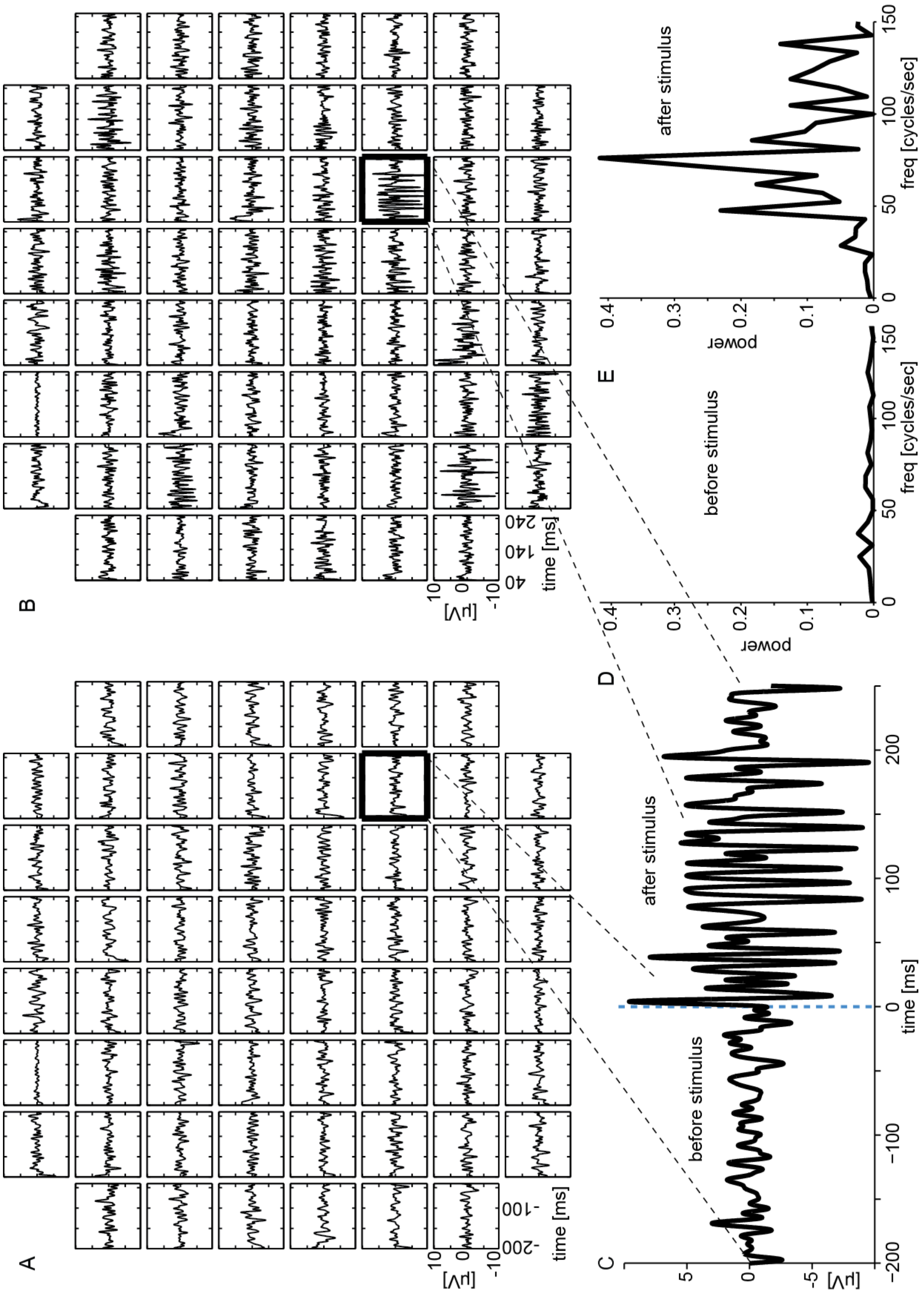

Figure S2. Light-evoked oscillatory activity is also reflected in single-trial raw voltage traces. Reminiscent of network rhythm signatures in local field potentials *in vivo* or in brain slices, the impact of brief (1msec) photoactivation was apparent also in single trials raw extracellular voltage recordings, low-pass filtered below 110 cycles/sec. For each MEA microelectrode, filtered voltage traces recorded ~200ms preceding (A) or following (B) the stimulus onset are displayed: the apparent transient increase in signal variance, recorded at most microelectrodes, correspond to reverberating network-wide spiking activity (Figs. 2A, 3). Estimating the power spectrum of a sample low-pass filtered raw trace (C-D) reveals light-induced oscillations in the same range as the spike PSTH (Fig. 3C), although with a much worst signal-to-noise ratio (Fig. 3C).
